# DPPA2/4 Promote the Pluripotency and Proliferation of Bovine Extended Pluripotent Stem Cells by Upregulating the PI3K/AKT/GSK3β/β-Catenin Signaling Pathway

**DOI:** 10.3390/cells13050382

**Published:** 2024-02-23

**Authors:** Shu Fang, Jing Wang, Guangbo Liu, Burong Qu, Jian Chunyu, Wenqiang Xu, Jinzhu Xiang, Xueling Li

**Affiliations:** The State Key Laboratory of Reproductive Regulation and Breeding of Grassland Livestock, Inner Mongolia University, Hohhot 010070, China; shike3302429@gmail.com (S.F.); nnlrl@mail.imu.edu.cn (J.W.); 32108121@mail.imu.edu.cn (G.L.); 32208050@mail.imu.edu.cn (B.Q.); 32008203@mail.imu.edu.cn (J.C.); xuwenqiang.btmc@gmail.com (W.X.)

**Keywords:** DPPA2, DPPA4, bEPSCs, reprogramming, pluripotency, proliferation, LEF1, PI3K/AKT pathway

## Abstract

Developmental pluripotency-associated 2 (DPPA2) and DPPA4 are crucial transcription factors involved in maintaining pluripotency in humans and mice. However, the role of DPPA2/4 in bovine extended pluripotent stem cells (bEPSCs) has not been investigated. In this study, a subset of bEPSC-related differentially expressed genes (DEGs), including *DPPA2* and *DPPA4*, was identified based on multiomics data (ATAC-seq and RNA-seq). Subsequent investigations revealed that double overexpression of DPPA2/4 facilitates the reprogramming of bovine fetal fibroblasts (BFFs) into bEPSCs, whereas knockout of DPPA2/4 in BFFs leads to inefficient reprogramming. DPPA2/4 overexpression and knockdown experiments revealed that the pluripotency and proliferation capability of bEPSCs were maintained by promoting the transition from the G1 phase to the S phase of the cell cycle. By activating the PI3K/AKT/GSK3β/β-catenin pathway in bEPSCs, DPPA2/4 can increase the nuclear accumulation of β-catenin, which further upregulates lymphoid enhancer binding factor 1 (LEF1) transcription factor activity. Moreover, DPPA2/4 can also regulate the expression of *LEF1* by directly binding to its promoter region. Overall, our results demonstrate that DPPA2/4 promote the reprogramming of BFFs into bEPSCs while also maintaining the pluripotency and proliferation capability of bEPSCs by regulating the PI3K/AKT/GSK3β/β-catenin pathway and subsequently activating LEF1. These findings expand our understanding of the gene regulatory network involved in bEPSC pluripotency.

## 1. Introduction

Extended pluripotent stem cells (EPSCs) can self-renew in culture and differentiate into both extraembryonic and embryonic lineages. Recently, human, pig, mouse, cow, and goat EPSCs have been established [1,2,3,4,5,6,7]. The initial culture system for EPSCs consists of recombinant human LIF, CHIR99021, minocycline hydrochloride, and (S)-(+)-dimethindene maleate, and this media is also known as LCDM. This culture system facilitated cultivation of EPSCs in the chemically defined medium without the need for overexpression of transgenes. Notably, our group successfully established bovine EPSCs (bEPSCs) using this culture system [6]. The bEPSCs displayed a compact and dome-like morphology and possessed extraembryonic and embryonic lineage differentiation ability when injected into mouse embryos. However, bovine EPSCs exhibit distinctive molecular characteristics, which suggests that the mechanisms responsible for maintaining bEPSC pluripotency are different from those of EPSCs from the other species [6]. Elucidating the signaling networks that govern self-renewal and pluripotency in bEPSCs is crucial for advancing the large-scale expansion of these cells and obtaining high-quality bovine pluripotent stem cells (bPSCs), which would enable the establishment of authentic bovine embryonic stem cells (bESCs).

Developmental pluripotency-associated 2 (DPPA2) and DPPA4 are crucial transcription factors involved in maintaining pluripotency in humans and mice [8,9]. DPPA2 and DPPA4 are specifically expressed in pluripotent cells, cancer cells, preimplantation embryos, and germline cells [9,10,11]. Both of these proteins have a conserved DNA-binding SAP (SAF-A/B, PIAS, and Acinus) motif and a conserved histone-binding C-terminal motif [9,12,13]. DPPA2/4 are actively involved in the regulation of pluripotent transformation [11,14,15] and play a pivotal role in modulating epigenetic marks during early differentiation [16,17]. The double overexpression of DPPA2/4 has been shown to cause upregulation of the γH2AX-mediated DNA damage response (DDR) pathway, resulting in improved reprogramming efficiency [15]. Additionally, it has been proposed that the molecular function of DPPA2/4 is in epigenetic priming, suggesting their role in establishing and accommodating the epigenome for facilitating subsequent cell differentiation [16,17]. However, the specific roles of DPPA2/4 in regulating the establishment and preservation of bEPSCs have not been determined.

In this study, we identified eight hub genes, including *DPPA2* and *DPPA4*, in EPSCs based on RNA-seq and ATAC-seq data. The regulatory effects of DPPA2 and DPPA4 on bEPSCs were investigated through a series of experiments, including overexpression, knockdown, knockout, and rescue experiments, which were coupled with transcriptional analyses during the establishment and maintenance of bovine EPSC pluripotency. Furthermore, the role of DPPA2 and/or DPPA4 in regulating the cell cycle was also investigated during the process of pluripotency maintenance. Subsequent studies confirmed that upregulation of DPPA2/4 activates the PI3K/AKT/GSK3β/β-catenin pathway, which upregulates the expression of the transcription factor LEF1. DPPA2/4 also directly modulates the expression of *LEF1* by binding to its promoter region. Thus, we describe a novel mechanism by which DPPA2/4 maintain the pluripotency and proliferation of PSCs.

## 2. Materials and Methods

### 2.1. Mice

C57 (ICR) mice and 5-week-old male NOD-SCID mice were obtained from Beijing Vital River Laboratory. C57ICR mice were used for the production of mouse embryonic fibroblasts (MEFs), which were used as feeder cells. Five-week-old male NOD-SCID mice were used for the teratoma experiments. The mice were individually housed under a 12-h light/dark cycle and provided unlimited access to food and water. All animal experiments were conducted in accordance with the guidelines of the Animal Protection and Utilization Committee and approved by the Inner Mongolia University Committee (approval code: IMU-MOUSE-2019-022, approval date: 26 August 2019).

### 2.2. Public Data Sources

The EPSC and fetal fibroblast (FF) RNA-seq and ATAC-seq data were obtained from the Gene Expression Omnibus (GEO) database (http://www.ncbi.nlm.nih.gov/geo, accessed on 10 May 2021). The details are shown in Supplementary Materials Additional File S2: Table S1.

### 2.3. RNA-Seq and ATAC-Seq Data Analysis

For RNA-seq, our previous studies described the detailed data processing procedures used for RNA-seq [6]. Briefly, clean reads were aligned with the reference genome using HISAT2 v2.0.5 [18]. Differential expression analysis was performed using the EDGER v3.34.1 [19] or limma R package v3.48.3 [20], and the parameters of |logFC| > 1 and *p* value < 0.05 were used as the screening criteria. For ATAC-seq, the raw reads were inspected with FastQC v0.11.9 for quality control. The clean reads were subsequently aligned to the reference genome (hg38) with Bowtie2 (v2.4.4) [21]. Peaks were annotated using the ChIPSeeker package v1.28.3 [22]. In addition, functional annotation of the genes was conducted using DAVID (*p* value < 0.05) [23], and volcano plots were generated using the ggplot2 package (http://ggplot2.org/, accessed on 15 August 2021). Heatmaps were created using the pheatmap package v1.0.12.

### 2.4. Reprogramming of Bovine Fetal Fibroblasts (BFFs)

PiggyBac(PB) transposons with CAG promoter, including PB-CAG-bovine OCT3/4, PB-CAG-bovine SOX2, PB-CAG-bovine KLF4, and PB-CAG-bovine c-MYC, were gifts from Xihe Li of Inner Mongolia University. The diagram of original PB vectors were shown by Zhao et al. and Wang et al. [24,25]. The piggyBac plasmids and piggyBac transposase vector were co-transfected into the BFFs (1 × 10^6^ cells) by electroporation. Reprogrammable BFFs (5000 cells per well) were then seeded into a 12-well feeder-seeded culture plate using Dulbecco’s modified Eagle’s medium (DMEM) supplemented with 10% fetal bovine serum. The medium was switched to the LCDM medium a day later. The cells were reprogrammed for a period of 7–22 days and subsequently maintained in the same medium supplemented with puromycin for an additional 4 days to select for fully reprogrammed bEPSCs. TrypLE Select was used to passage the colonies. Flow cytometry was used to sort GFP+ cells, which had been successfully transfected with PB-CAG-bovine OCT3/4/SOX2/KLF4/c-MYC plasmids and PB-CAG-GFP (bEPSCs^C^) or PB-CAG-bovine OCT3/4/SOX2/KLF4/c-MYC and PB-CAG-bovine DPPA2/DPPA4 containing green fluorescent protein gene plasmids (bEPSCs^A2/4^). To determine their pluripotency status, the cells were immunostained with antibodies against NANOG, OCT4, SOX2, SSEA1, and SSEA4.

### 2.5. Bovine EPSC Culture

The bovine EPSCs were maintained on mitomycin C-treated MEF feeder cells in LCDM in 5% CO_2_ at 38.5 °C as previously described [1]. The LCDM medium contained equal amounts of DMEM/F12 (11330-033, Gibco, New York, NY, USA), Neurobasal (21103-049, Gibco, New York, NY, USA), 0.5% N2 supplement (17502-048, Gibco, New York, NY, USA), 1% B27 supplement (17504-044, Gibco, New York, NY, USA), 1% nonessential amino acids (M7145, Sigma-Aldrich, St. Louis, MO, USA), 1% L-glutamine (Sigma-Aldrich, St. Louis, MO, USA), 0.1 mM β-mercaptoethanol (Sigma-Aldrich, St. Louis, MO, USA), 1% penicillin/streptomycin (15140122, Gibco, New York, NY, USA), and 5% knockout serum replacement (10828028, Gibco, New York, NY, USA). Small molecules and cytokines were added to the LCDM medium at the following final concentrations: 1 mM CHIR99021 (HY-10182, MCE, Monmouth Junction, NJ, USA), 2 mM (S)-(+)-dimethindene maleate (1425, R&D Systems, Minneapolis, MN, USA) and 2 mM minocycline hydrochloride (HY-17412, MCE, Monmouth Junction, NJ, USA) and 10 ng/mL recombinant human LIF (300-05, Peprotech, Cranbury, NJ, USA). The cells were passaged with TrypLE Select (12563-029; Gibco, New York, NY, USA), and the medium was changed every day. In addition, to investigate the PI3K/AKT pathway in bEPSCs treated with the PI3K inhibitor LY294002 (20 μM, MCE) alone or in combination with DPPA2/4 double overexpression; dimethyl sulfoxide (DMSO) was used as a vehicle for the negative control assays.

### 2.6. Knockout and Overexpression of DPPA2/4

For the generation of sgRNA vectors, we inserted the amplified fragments into the pSpCas9(BB)-2A-Puro (PX459) vector, which were provided by X. Li [26]. The sgRNA sequences used to target DPPA2 and DPPA4 were provided in Supplementary Materials Additional File S2: Table S2. For overexpression, we constructed the PB vectors of DPPA2 and DPPA4, PB-CAG-bovine DPPA2 and PB-CAG-bovine DPPA4, in which cDNAs of DPPA2 and DPPA4 were linked to GFP by T2A, respectively, and were under control of the CAG promoter. The overexpression vectors were cotransfected into BFFs (1 × 10^6^ cells) with OSKM and bEPSCs (1 × 10^7^ cells), and the knockout vectors were cotransfected into BFFs (1 × 10^6^ cells) with OSKM via electroporation and replated on mitomycin C-treated MEFs for 48 h. The cells were selected with puromycin (1 μg/mL) for 2 days, the GFP+ cells (cells that had been successfully transfected with PB transposase and PB-CAG-bovine DPPA2/4 plasmids containing green fluorescent protein) were sorted by flow cytometry, and 1 × 10^4^ cells were plated onto a 10 cm dish with mitomycin C-treated MEFs. Then, monoclonal bEPSCs were established. Generation of cells with the correct modifications were validated by PCR and DNA sequencing. Protein expression was analyzed by Western blotting. The primers used for DPPA2/4 CDS amplification were provided in Supplementary Materials Additional File S2: Table S3.

### 2.7. Small Interfering RNA (siRNA) Transfection

Bovine EPSCs were grown to 75% confluence. The cells were transfected with DPPA2/4- or LEF1-specific siRNAs or with nontargeting siRNA as a negative control using GP-transfect-Mate (230228, GenePharma, Suzhou, China) transfection reagent. After 24 h, the transfection mixture was replaced with LCDM, and the cells were maintained in LCDM. The siRNA sequences used to target DPPA2, DPPA4, and LEF1 were provided in Supplementary Materials Additional File S2: Table S4.

### 2.8. AP Activity Assay

We performed AP staining using an Alkaline Phosphatase Staining Kit (C3206, Beyotime Biotechnology, Shanghai, China) according to the manufacturer’s instructions. Briefly, bovine EPSCs were fixed in 4% paraformaldehyde (PFA) for 10 min, incubated in AP staining reagent for 30 min at room temperature (RT) in the dark, washed with PBS and visualized using a microscope (Nikon; Tokyo, Japan).

### 2.9. Chromosome Number Analysis

The EPSCs were subjected to treatment with KaryoMAX colcemid solution (Gibco) for 3 h. Subsequently, the samples were incubated in 0.075 M KCl at 37 °C for a period of 30 min. The resulting cell pellets were fixed in a solution consisting of cold glacial acetic acid and methanol at a 1:3 ratio for 40 min. The fixed cells were subsequently subjected to centrifugation at 200× *g* for 5 min. The cells were then transferred to cold slides and dried at room temperature (RT). Finally, the samples were stained with Giemsa dye, and images were acquired using a Nikon Eclipse 80i microscope (Nikon). A minimum of 50 metaphases were analyzed for each sample.

### 2.10. In Vitro Differentiation

To differentiate bEPSCs into embryoid bodies (EBs), cells were dissociated using TrypLE Select and then cultured in IMDM (12440-053; Gibco) supplemented with 15% FBS (Bovogen, Keilor East, Australia) in 5% CO_2_ at 38.5 °C. After 4–6 days, EBs were collected and cultivated on 4-well plates coated with matrigel. The culture medium was replaced every two days, and the cells were cultured for 10–15 days. We conducted immunofluorescence and qRT-PCR experiments on the EB samples separately.

### 2.11. Teratoma Formation

Bovine EPSCs were dissociated and suspended in DPBS at a concentration of 2 × 10^7^ cells/mL. Subsequently, a total of 5 × 10^6^ cells were subcutaneously injected into 5-week-old male NOD-SCID mice. After a period of 15–20 weeks, teratomas were isolated, fixed overnight with 4% PFA in PBS, and stained with hematoxylin and eosin (HE) for analysis.

### 2.12. Immunofluorescence Staining

Cells were fixed with 4% PFA for 30 min at RT and then blocked in a solution of DPBS containing 10% goat serum and 0.5% Triton X-100 for 2 h at RT. Subsequently, the slides were stained with primary antibodies at a dilution ratio of 1:100 overnight. Afterward, the slides were washed three times with DPBS, incubated with secondary antibodies for 1 h at RT, and finally were washed with DPBS. The nuclei were stained with DAPI for 5 min at RT and then cover-slipped and imaged using a Nikon forward laser confocal microscope (Nikon; Tokyo, Japan). The primary antibodies used were as follows: anti-DPPA2 (ab91318; Abcam, Cambridge, UK), anti-DPPA4 (ab154642; Abcam, Cambridge, UK), anti-OCT4 (sc-9081; Santa Cruz Biotechnology, Shanghai, China), anti-SOX2 (4900s; Cell Signaling Technology, Boston, MA, USA), anti-NANOG (500-P236; PeproTech, London, UK), anti-GFAP (Z0334; Dako, Carpinteria, CA, USA), anti-AFP (MAB1368; R&D Systems, Minneapolis, MN, USA), anti-α-SMA (ab5694; Abcam, Cambridge, UK), anti-CDX2 (MU392A-UC; Biogenex, San Francisco, CA, USA), anti-SSEA1 (MAB4301; Santa Cruz Biotechnology, Dallas, TX, USA), and anti-SSEA4 (MAB4304; Santa Cruz Biotechnology, Dallas, TX, USA). The secondary antibodies used were as follows: Alexa594-conjugated donkey anti-mouse IgG (Invitrogen, Waltham, MA, USA), goat anti-mouse IgG and IgM (AP130F; Millipore, Burlington, MA, USA) and Alexa488-conjugated goat anti-rabbit IgG (A-21206; Life Technologies, Carlsbad, CA, USA).

### 2.13. Quantitative RT-PCR

Total RNA was extracted using TRIzol and quantified using a Nanodrop (Thermo Fisher Scientific, Waltham, MA, USA). cDNAs were synthesized using the PrimeScript^RT^ Reagent Kit with gDNA Eraser for qPCR from Takara. qRT-PCR was performed in an ABI 7500 real-time PCR system from Applied Biosystems using the GoTaq^®^ qPCR Master Mix from Promega following the manufacturer’s instructions. The relative expression levels were analyzed using the 2^−ΔΔCt^ method and normalized to *GAPDH* expression. The primers used for qRT-PCR analyses are provided in Supplementary Materials Additional File S2: Table S5.

### 2.14. Western Blotting and Antibodies

Cells were washed with ice-cold DPBS and then incubated with 1× KGP250 lysis buffer (KeyGEN BioTECH, Nanjing, China) supplemented with 100 mM PMSF, protease, and phosphatase inhibitors for 20 min on ice. The lysates were centrifuged at 13,000× *g* at 4 °C for 15 min, after which the protein concentration was determined using a BCA protein assay (KeyGEN BioTECH). A total of 30–60 μg of protein lysate was separated via SDS-PAGE and transferred to a polyvinyl difluoride (PVDF) membrane from Pall. The blots were then blocked with 5% milk in TBS-T buffer and incubated overnight at a dilution ratio of 1:1000 with primary antibodies. After washing with DPBS, the blots were incubated with secondary antibodies for 1 h at RT, which was followed by additional washing steps. Finally, the blots were developed using the SuperSignal West Pico Chemiluminescent Substrate from Pierce. The antibodies used in this study were as follows: anti-GAPDH (10494-1-AP; Proteintech, Franklin Lakes, NJ, USA), anti-DPPA2 (ab91318; Abcam, Cambridge, UK), anti-DPPA4 (ab154642; Abcam, Cam-bridge, UK), anti-OCT4 (sc-9081; Santa Cruz Biotechnology, Shanghai, China), an-ti-SOX2 (4900s; Cell Signaling Technology, Boston, MA, USA), anti-NANOG (500-P236; PeproTech), anti-DNMT3A (D23G1; Cell Signaling Technology, Boston, MA, USA), anti-DNMT3B (bs-0301R; Bioss, Beijing, China), anti-LEF1 (abs137050; Absin, Shanghai, China), anti-AKT (60203-2-Ig; Proteintech Technology, Wuhan, China), phospho-AKT (4060P; Cell Signaling Technology, Boston, MA, USA), anti-PI3K (#T40064; Abmart, Shanghai, China), phospho-PI3K (TA3242S; Abmart, Shanghai, China), anti-GSK3B (22104-1-AP; Proteintech Biotechnology, Wuhan, China), phospho-GSK3B (Ser9) (675-1-Ig; Proteintech Biotechnology, Wuhan, China), phospho-beta catenin (Ser675) (28853-1-AP; Proteintech Biotechnology, Wuhan, China) and anti-β-catenin (#8480; Cell Signaling Technology, Boston, MA, USA).

### 2.15. Cell Viability Assay

Approximately 1000 bEPSCs were seeded onto 96-well plates using 100 μL of LCDM. Cell viability was assessed at specified time points (24 h, 48 h, 72 h, and 96 h) using a CCK-8 kit (Sigma) in accordance with the manufacturer’s instructions. Briefly, 10 μL of CCK-8 solution in 100 µL of LCDM was added to each well, and subsequently, the optical density (OD) at 450 nm was measured using a microplate reader (Thermo, Waltham, MA, USA).

### 2.16. EdU Cell Proliferation Assay

EdU (5-ethynyl-2′-deoxyuridine) is a novel thymidine (thymidine deoxyriboside) analog that can be incorporated into newly synthesized DNA during DNA synthesis. Cell proliferation was analyzed as previously described [27] using a commercially available EdU kit (BeyoClick™ EdU Cell Proliferation Kit with Alexa Fluor 555; Beyotime) according to the manufacturer’s instructions. EdU-positive cell images from the different treatment groups were obtained using a Nikon AX forward laser confocal microscope system (Nikon; Tokyo, Japan).

### 2.17. Flow Cytometry Assay

The cells were dissociated into single cells and sorted by a BD Aria instrument. For determination of cell cycle distribution, cells were fixed in 70% ethanol and stained with propidium iodide (PI) and RNase in the dark at RT for 30 min. Then, the samples were inspected via a Cytoflex LX flow cytometer.

### 2.18. ChIP-qPCR

ChIP experiments were performed with anti-DPPA2 and anti-DPPA4 antibodies. Briefly, a total of 6 × 10^7^ bEPSCs were fractionated, and the purified nuclei were sonicated to generate chromatin fragments ranging from 200 to 600 bp in size. These purified chromatin fragments were then subjected to overnight incubation with anti-DPPA2 and anti-DPPA4 antibodies. The DNA fragments bound to the antibodies were pulled down using Protein G magnetic beads and subsequently purified for RT-PCR assays. Each experiment was performed at least three times, and each experiment included three technical replicates. Statistical analyses were conducted using a t test, and statistical significance was determined with a *p* value of 0.05 for each experiment. The primers used for ChIP-qPCR analyses are provided in Supplementary Materials Additional File S2: Table S6.

### 2.19. RNA Sequencing

RNA-seq experiments were carried out on Control^OE^ and DPPA2/4^OE^ bEPSCs, as well as on Control^KD^ and DPPA2/4^KD^ bEPSCs. Total RNA was extracted from the respective groups using the previously described method. Subsequently, the RNA was converted into a template molecule library using the NEBNext^®^ Ultra^TM^ RNA Kit for sequencing on the Illumina HiSeq platform (Novogene Co., Ltd., Cambridge, UK). The resulting raw reads were analyzed using the “RNA-seq and ATAC-seq data analysis” approach described earlier.

### 2.20. Statistical Analysis

All the experiments were performed with at least 3 biological and technical replicates. Graphical presentation and statistical analysis of the data were performed with GraphPad Prism 8.0. The data are presented as the means ± SDs, and statistical significance was determined with Student’s two-tailed *t* test: * *p* < 0.05; ** *p* < 0.01; *** *p* < 0.001.

## 3. Results

### 3.1. Using RNA-Seq and ATAC-Seq Data Identify DPPA2 and DPPA4 as Hub Genes Involved in EPSC Reprogramming

**Figure 1:**
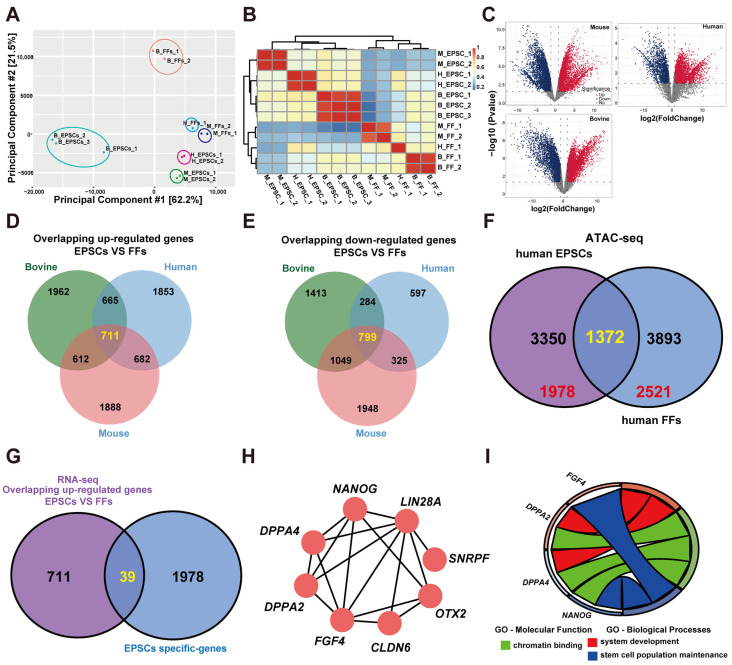
Identification of hub genes involved in EPSC reprogramming. (**A**) Principal component analysis (PCA) of the RNA-seq data between FFs and EPSCs. (**B**) Heatmap showing the correlation of the samples. (**C**) The differential gene expression analysis of human, mouse, and bovine. Significantly differentially expressed peaks are denoted by red and blue dots. (**D**,**E**) Venn diagrams showing the overlap of differentially upregulated (**D**) and downregulated (**E**) genes. (**F**) The Venn diagram shows genes with open chromatin in human EPSCs and human FFs. (**G**) The number of overlapping genes between up-regulated genes (RNA-seq) and genes with open chromatin in EPSCs only (ATAC-seq). (**H**) Eight hub genes were selected by the cytoHubba plugin in Cytoscape. (**I**) GO-molecular function analysis revealed that hub gene *FGF4*, *DPPA2*, *DPPA4*, and *NANOG* were significantly associated with chromatin binding (*p* value < 0.05). GO biological process analysis revealed that these hub genes were enriched mainly in system development and stem cell population maintenance.

To identify pivotal biomarkers associated with EPSC reprogramming, we conducted a comprehensive analysis of gene expression and chromatin properties using the published data of RNA-seq and ATAC-seq, respectively. First, RNA-seq analysis was performed on fetal fibroblasts (FFs) and EPSCs derived from mice, humans, and bovines, as described in published data. Principal component analysis (PCA) (Figure 1A) and sample correlation (Figure 1B) demonstrated that there were significant differences in the expression patterns between EPSCs and FFs. A total of 8014 DEGs were found between EPSCs and FFs in mice, 5916 were found in humans, and 6495 were found in bovines (Figure 1C). Subsequently, we selected the overlapping DEGs for further scrutiny. As shown in Figure 1D,E, we obtained 711 upregulated overlapping DEGs and 799 downregulated overlapping DEGs.

Next, published data were used to analyze the chromatin accessibility profiles of FFs and EPSCs. Unfortunately, no chromatin property data (ATAC-seq) were found for mice or bovines by searching the GEO database. Through investigation of chromatin property data in humans, we found a total of 3350 and 3893 genes with open chromatin in human EPSCs and human FFs, respectively (Figure 1F). Among them, 1978 and 2521 genes with open chromatin in EPSCS and FF only, respectively (Supplementary Materials Additional File S1). Further investigation revealed 39 overlapping genes when comparing the upregulated genes and the genes linked to open chromatin in EPSCs (Figure 1G). Subsequently, we utilized CytoHubba and identified 8 hub genes (*DPPA2*, *DPPA4*, *NANOG*, *LIN28A*, *SNRPF*, *OTX2*, *FGF4*, and *CLDN6*); interestingly, *DPPA2* and *DPPA4* were among them (Figure 1H). To further understand the functional roles of these hub genes (8 genes), Gene Ontology (GO) analysis was conducted. GO-molecular function analysis revealed that these hub genes were significantly associated with chromatin binding (*p* value < 0.05) (Figure 1I). GO biological process analysis revealed that these hub genes were enriched mainly in system development and stem cell population maintenance (*p* value < 0.05) (Figure 1I). The above results implied that these hub genes including *DPPA2* and *DPPA4* may play a critical role in EPSC reprogramming. 

### 3.2. DPPA2 and DPPA4 Are Essential for Establishing Bovine Extended Potential Stem Cells by Reprogramming

**Figure 2:**
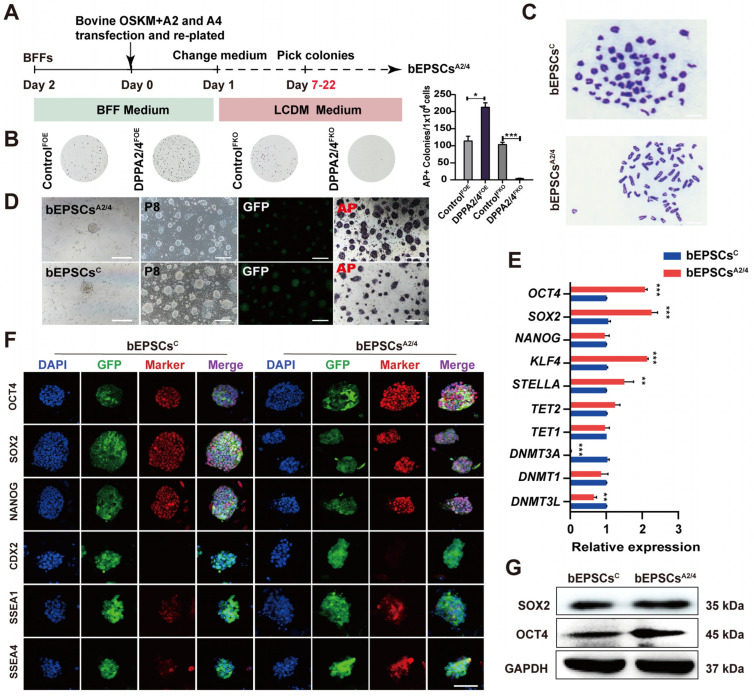
Reprogramming of BFFs into bEPSCs requires DPPA2 and DPPA4. (**A**) Experimental strategy for generating bovine EPSCs from BFFs. (**B**) AP staining of reprogrammed BFFs from the control, DPPA2/4-double overexpressing, and DPPA2/4-double knockout groups. (**C**) Karyotype analysis of bEPSCs^C^ and bEPSCs^A2/4^. (**D**) AP staining for bEPSCs^C^ and bEPSCs^A2/4^. GFP (bEPSCs^A2/4^) in the upper panel represents cell colonies successfully transfected by PB-CAG-bovine DPPA2/4, while GFP (bEPSCs^C^) in the lower panel represents the control group cell colonies successfully transfected by PB-CAG-GFP. (**E**) qRT-PCR analysis of key endogenous pluripotency genes in bEPSCs^A2/4^ and bEPSCs^C^. (**F**) Immunostaining of OCT4, SOX2, NANOG, CDX2, SSEA1, and SSEA4 in bEPSCs^C^ and bEPSCs^A2/4^ cells. Scale bar, 50 μm. GFP in the left panel represents the control group cell clones successfully transfected with PB-CAG-GFP, whereas the GFP in the right panel represents cell clones successfully transfected with PB-CAG-bovine DPPA2/4. (**G**) Protein levels of OCT4 and SOX2 in bEPSCs^C^ and bEPSCs^A2/4^; GAPDH served as a loading control. The data are presented as the means ± SDs; n = 3 independent experiments (* *p* < 0.05; ** *p* < 0.01; *** *p* < 0.001).

Previous studies have shown that double overexpression of DPPA2/4 enhances the generation of induced pluripotent stem cells (iPSCs) and accelerates the transition to pluripotency [15,28]. However, the role of DPPA2/4 in the establishment of bEPSCs has not yet been explored. Four exogenous reprogramming factors, bOSKM (bovine OCT4, SOX2, KLF4, and c-MYC), were used to reprogram BFFs. To determine the importance of DPPA2 and DPPA4 in the reprogramming of BFFs, we performed DPPA2/4-single and double overexpressing and knockout (DPPA2^FOE^, DPPA4^FOE^, DPPA2/4^FOE^, DPPA2^FKO^, DPPA4^FKO^, and DPPA2/4^FKO^) during the reprogramming process (Supplementary Materials Additional File S2: Figure S1A). The piggyBac and CRISPR/Cas9 systems were used to engineer recombinant DPPA2/4 plasmids for both overexpression and knockout purposes. These plasmids were subsequently transfected into BFFs with bOSKM (Figure 2A). The results showed that single and double knockout of DPPA2/4 prevents BFF reprogramming into bEPSCs, whereas double overexpression of DPPA2/4 significantly increases the number of alkaline phosphatase (AP)-positive colonies during reprogramming, and there was no notable increase in colony numbers observed upon DPPA2 and DPPA4 individual overexpression (Figure 2B, Supplementary Materials Additional File S2: Figure S1B). Furthermore, we found that the derived bEPSCs maintained an undifferentiated state and normal karyotype (2n = 60) even after being passaged for more than 40 generations (Figure 2C).

**Figure 3:**
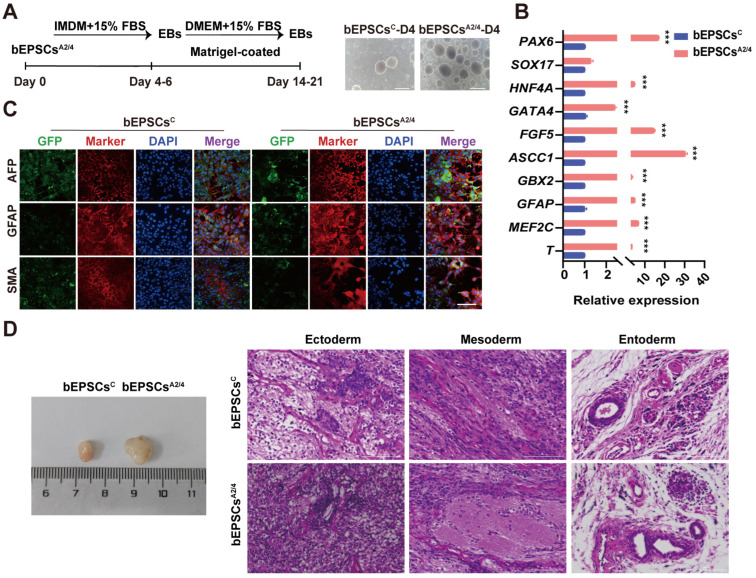
bEPSCs^A2/4^ cells exhibited greater differentiation ability than control bEPSCs^C^ cells. (**A**) A schematic illustration of the generation of EBs from bEPSCs^C^ and bEPSCs^A2/4^ and the morphologies of the EBs. Scale bar, 100 μm. (**B**) qRT-PCR analysis of EBs at day 20. (**C**) Immunostaining of AFP, GFAP, and SMA in the EBs derived from bEPSCs^C^ and bEPSCs^A2/4^ cells at day 20. Scale bar, 100 μm. (**D**) Teratoma derived from bEPSCs^C^ and bEPSCs^A2/4^ (passage 35). HE staining analysis revealed the presence of the three germ layers (mesoderm, ectoderm, and endoderm). Scale bar, 50 μm. The data are presented as the means ± SDs; n = 3 independent experiments (*** *p* < 0.001).

Next, we compared the characteristics of bEPSCs obtained through DPPA2/4 double overexpression (bEPSCs^A2/4^) with those of control bEPSCs (bEPSCs^C^). The results showed that bEPSCs^A2/4^ exhibited a more homogeneous cell population and clear domed colony morphology compared with bEPSCs^C^; however, both cell types exhibited strong AP-positive staining (Figure 2D). To investigate the expression differences of bOSKM between bEPSCs^C^ and bEPSCs^A2/4^, we designed specific primers to distinguish endogenous and exogenous bOSKM and employed qRT-PCR on these cell lines. The findings revealed that bEPSCs^C^ and bEPSCs^A2/4^ displayed elevated levels of endogenous bOSKM expression in comparison to BFFs, along with dramatically reduced expression of exogenous bOSKM. Furthermore, bEPSCs^A2/4^ exhibited high levels of endogenous *DPPA2* and *DPPA4* expression, with low expression of exogenous *DPPA2* and *DPPA4* (Supplementary Materials Additional File S2: Figure S1C). The residual expression of exogenous genes may be due to the strong CAG promoter. Compared with bEPSCs^C^ cells, bEPSCs^A2/4^ expressed higher levels of endogenous pluripotency genes, including *STELLA*, *OCT4 (POU5F1)*, *KLF4*, and *SOX2*, but they exhibited significantly lower expression of *DNMT3A* and *DNMT3L* (Figure 2E). Immunofluorescence analyses demonstrated the positive expression of the pluripotency markers OCT4, SOX2, KLF4, c-MYC, NANOG, SSEA-1, and SSEA-4 in both bEPSCs^A2/4^ and bEPSCs^C^ (Figure 2F), and Western blotting also confirmed the up-regulation of OCT4 and SOX2 (Figure 2G). Notably, these cells did not express the lineage marker CDX2 (Figure 2F). To assess the ability of bEPSCs^C^ and bEPSCs^A2/4^ cells to generate germ layer derivatives in vitro and in vivo, the expression patterns of lineage marker genes were examined using qRT-PCR and immunofluorescence (Figure 3A–C). The results revealed that the EBs derived from bEPSCs^A2/4^ cells exhibited higher expression levels of lineage marker genes (*T*, *MEF2C*, *GFAP*, *GBX2*, *ASCC1*, *FGF5*, *GATA4*, *HNF4A*, and *PAX6*) than did bEPSCs^C^ cells (Figure 3B). Moreover, in vivo, both bEPSCs^C^ and bEPSCs^A2/4^ successfully formed teratomas, which contain cell types that originate from three germ layers. Importantly, bEPSCs^A2/4^ formed larger teratomas than bEPSCs^C^ did (Figure 3D). Taken together, these findings indicate that DPPA2/4 are vital for the reprogramming of BFFs into bEPSCs and that the double overexpression of DPPA2/4 enhances reprogramming.

### 3.3. DPPA2/4 Knockdown Reduces Pluripotency and Accelerates Early Differentiation of Established bEPSCs

**Figure 4:**
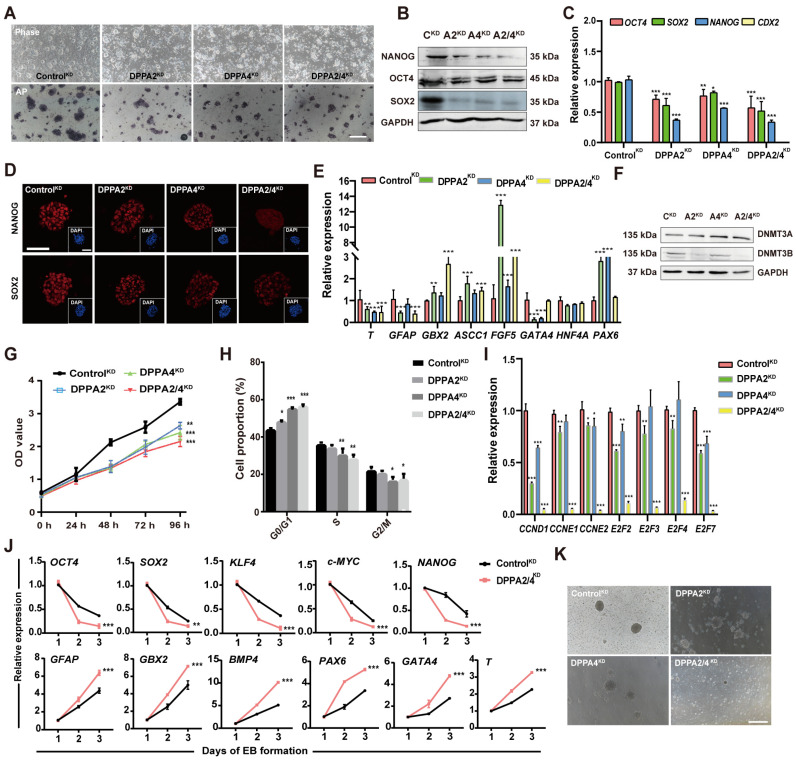
DPPA2/4 knockdown affects the pluripotency and early differentiation of bEPSCs. (**A**) Morphology and AP staining of Control^KD^, DPPA2^KD^, DPPA4^KD^, and DPPA2/4^KD^ bEPSCs. Scale bar, 50 μm. (**B**) Protein levels of OCT4, SOX2, and NANOG in Control^KD^, DPPA2^KD^, DPPA4^KD^, and DPPA2/4^KD^ bEPSCs. (**C**) qRT-PCR analysis of *OCT4*, *SOX2, NANOG*, and *CDX2*. (**D**) Immunofluorescence staining for SOX2 and NANOG in Control^KD^, DPPA2^KD^, DPPA4^KD^, and DPPA2/4^KD^ bEPSCs. Scale bars, 50 µm. (**E**) qRT-PCR analysis of *T*, *GFAP*, *GBX2*, *ASCC1*, *FGF5*, *GATA4*, *HNF4A*, and *PAX6*. (**F**) Western blotting analysis of DNMT3A and DNMT3B protein expression. (**G**) Proliferation of Control^KD^, DPPA2^KD^, DPPA4^KD^, and DPPA2/4^KD^ bEPSCs. (**H**) Cell cycle analysis of Control^KD^, DPPA2^KD^, DPPA4^KD^, and DPPA2/4^KD^ bEPSCs. (**I**) qRT-PCR analysis of cell cycle regulatory genes. (**J**) qRT-PCR was used to detect changes in the expression levels of pluripotent and differentiation marker genes at 1–3 days. (**K**) EB morphology of Control^KD^, DPPA2^KD^, DPPA4^KD^, and DPPA2/4^KD^ bEPSCs (n = 3). Scale bars, 100 µm. The data are presented as the means ± SDs; n = 3 independent experiments (* *p* < 0.05; ** *p* < 0.01; *** *p* < 0.001).

To investigate the role of DPPA2/4 in maintaining the pluripotency of established bEPSCs, we performed DPPA2/4 knockdown experiments; specifically, we used small interfering RNA (siRNA) to reduce the expression of DPPA2 (DPPA2^KD^), DPPA4 (DPPA4^KD^), and both DPPA2 and DPPA4 (DPPA2/4^KD^) in bEPSCs. The knockdown effects were confirmed by qRT-PCR, Western blotting, and immunofluorescence (Supplementary Materials Additional File S2: Figure S2A–C). Single and double knockdown of DPPA2 and DPPA4 resulted in the formation of flattened and loose colonies, and the colonies displayed blurred boundaries compared with the control cells (Figure 4A, Supplementary Materials Additional File S2: Figure S2D). Moreover, the colonies of knockdown cells exhibited weak and heterogeneous AP staining, which indicated a diminished self-renewal capacity. Consistent with the AP staining results, concurrent knockdown of DPPA2/4 in bEPSCs reduced the expression levels of NANOG, OCT4, and SOX2 at both the mRNA and protein levels (Figure 4B–D, Supplementary Materials Additional File S2: Figure S2E), whereas double knockdown of DPPA2/4 was accompanied by an increase in the expression of some embryonic and extraembryonic lineage genes, such as *GBX2*, *ASCC1*, and *FGF5*. The expression of *PAX6* was higher in bEPSCs with either DPPA2 or DPPA4 knockdown, potentially suggesting the differentiation of bEPSCs (Figure 4E). However, after DPPA2/4 single or double knockdown, the bEPSCs still maintained normal chromosome numbers throughout successive passages (Supplementary Materials Additional File S2: Figure S2F). We performed qRT-PCR analysis to compare the expression of naïve and primed pluripotency genes among Control^KD^, DPPA2^KD^, DPPA4^KD^, and DPPA2/4^KD^ bEPSCs. As expected, bEPSCs knockdown of DPPA2/4 exhibited substantial alteration of both naïve and primed genes (Supplementary Materials Additional File S2: Figure S2G,H). The alteration of DNMT3A was further confirmed at the protein level (Figure 4F). Furthermore, DPPA2^KD^, DPPA4^KD^, and DPPA2/4^KD^ bEPSCs exhibited a diminished proliferation ability (Figure 4G). Cell cycle analysis revealed that cells’ double knockdown of DPPA2/4 had a significantly extended G0/G1 phase and a shortened S phase (Figure 4H, Supplementary Materials Additional File S2: Figure S2I). Furthermore, qRT-PCR analysis of DPPA2/4 double knockdown cells demonstrated the differential expression of numerous key regulatory factors involved in the cell cycle and apoptosis-related genes (Figure 4I, Supplementary Materials Additional File S2: Figure S2J).

The effect of DPPA2/4 single and double knockdown on the differentiation of bEPSCs was further examined by inducing the formation of EBs. The expression of marker genes was analyzed after three days of bEPSC differentiation. The qRT–PCR results indicated that the decrease in pluripotency-related genes and the upregulation of differentiation genes were accelerated in the double knockdown group compared to the control group, which suggested an increase in the differentiation rate (Figure 4J). DPPA2/4 double knockdown bEPSCs resulted in the formation of smaller and looser EBs, and this was accompanied by a reduction in EB number (Figure 4K). Moreover, the mRNA levels of the marker genes for the ectoderm (*GFAP*), mesoderm (*FGF5*), and endoderm (*HNF4A*) were decreased in the EBs derived from DPPA2/4 double knockdown bEPSCs (Supplementary Materials Additional File S2: Figure S2K). Consistently, the expression of the GFAP (ectoderm), SMA (mesoderm), and AFP (endoderm) marker proteins was hardly detectable in these cells (Supplementary Materials Additional File S2: Figure S2L). These findings suggested that single and double knockdown of DPPA2/4 may promote early differentiation indicated by *GATA4* and *SOX17* and impair late differentiation indicated by AFP in the EBs derived from bEPSCs. Thus, DPPA2/4 clearly play a crucial role in maintaining pluripotency in bEPSCs, and impairment of DPPA2/4 affects the pluripotency and differentiation of bEPSCs.

### 3.4. Overexpression of DPPA2 and DPPA4 Increases the Pluripotency of Established bEPSCs and Promotes Their Proliferation

**Figure 5:**
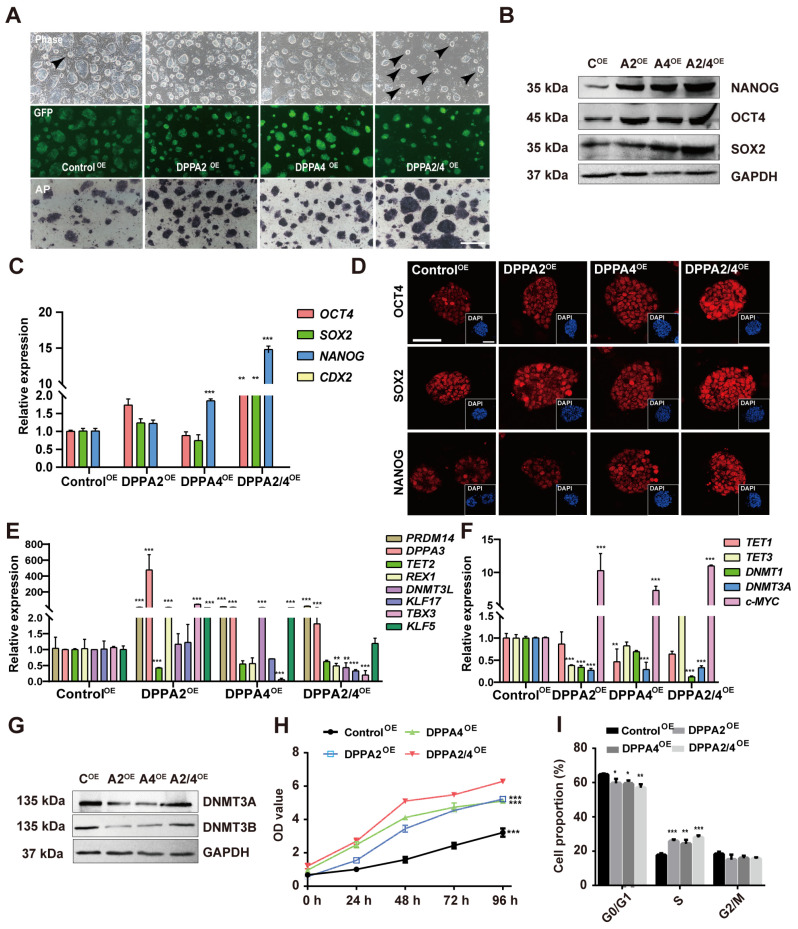
Overexpression of DPPA2 and DPPA4 increases the pluripotency of bEPSCs and promotes their proliferation. (**A**) AP staining of Control^OE^, DPPA2^OE^, DPPA4^OE^, and DPPA2/4^OE^ bEPSC colonies cultured for 3 days. Scale bars, 100 µm. GFP indicates the successfully transfected cells. The black arrow indicates the "dome" form of bEPSCs. (**B**) Protein levels of OCT4, SOX2, and NANOG in different treatment groups of bEPSCs. (**C**) qRT-PCR analysis of pluripotency marker genes and trophectoderm marker genes. (**D**) Immunofluorescence staining for OCT4, SOX2, and NANOG in Control^OE^, DPPA2^OE^, DPPA4^OE^, and DPPA2/4^OE^ bEPSCs. Scale bars, 50 µm. (**E**,**F**) qRT-PCR analysis of naïve and primed marker genes. (**G**) Western blotting analysis of DNMT3A and DNMT3B protein expression in different treatment groups of bEPSCs. (**H**) The proliferation rates of Control^OE^, DPPA2^OE^, DPPA4^OE^, and DPPA2/4^OE^ bEPSCs. (**I**) Cell cycle analysis of Control^OE^, DPPA2^OE^, DPPA4^OE^, and DPPA2/4^OE^ bEPSCs. The data are presented as the means ± SDs; n = 3 independent experiments (* *p* < 0.05; ** *p* < 0.01; *** *p* < 0.001).

Previous studies have demonstrated that the double overexpression of DPPA2/4 expedites the reprogramming of cells to a pluripotent state and induces swift modifications in chromatin [15,16,17]. Additionally, double overexpression of DPPA2/4 is linked to facilitation of the 2C-like transcriptional program through the modulation of Dux, suggesting that these proteins are involved in various functional roles [14,15,28,29]. To explore the function of DPPA2/4 in bEPSCs, we overexpressed DPPA2 and/or DPPA4 by transfecting the piggyBac vector into bEPSCs; the derived cells were named Control^OE^, DPPA2^OE^, DPPA4^OE^, and DPPA2/4^OE^. Following transfection, a notable increase in the expression of DPPA2/4 was observed in DPPA2/4^OE^ cells (Supplementary Materials Additional File S2: Figure S3A–C). The impact of DPPA2 and DPPA4 on the self-renewal of bEPSCs was determined by AP staining, as depicted in Figure 5A. After overexpressing, whether expressed individually or in combination, the bEPSCs exhibited undifferentiated morphology and high AP activity (Figure 5A). Furthermore, concurrent overexpression of DPPA2/4 induced a more pronounced “domed” morphology in bEPSCs (as indicated by black arrows) (Figure 5A), and DPPA2/4^OE^ bEPSCs expressed elevated levels of OCT4, SOX2, and NANOG mRNA and protein (Figure 5B,C). Immunostaining confirmed the upregulation of the OCT4, SOX2, and NANOG proteins in DPPA2/4^OE^ bEPSCs (Figure 5D).

Additionally, a small number of SSEA1- and SSEA4-expressing cells were observed among the DPPA2/4^OE^ bEPSCs, while CDX2 was not detected (Supplementary Materials Additional File S2: Figure S3D). Double overexpression of DPPA2/4 in bEPSCs led to a significant increase in the mRNA levels of some naïve pluripotency genes, such as *PRDM14* and *DPPA3*, while the mRNA level of the other gene such as *REX1*, *DNMT3L*, *KLF17*, and *TBX3* was decreased (Figure 5E). However, the mRNA levels of primed pluripotency genes, including *DNMT1* and *DNMT3A*, were significantly reduced (Figure 5F). Western blotting also demonstrated a significant decrease in DNMT3A and DNMT3B following separate overexpression of DPPA2 or DPPA4 in bEPSCs (Figure 5G). Moreover, the results of the cell proliferation experiment clearly demonstrated that DPPA2^OE^, DPPA4^OE^, and DPPA2/4^OE^ bEPSCs exhibited significantly greater proliferation rates (Figure 5H). Cell cycle analysis revealed a significant decrease in the proportion of bEPSC-overexpressing cells in the G0/G1 phase and a significant increase in the proportion of cells in the S phase compared to those in the control cells (Figure 5I, Supplementary Materials Additional File S2: Figure S3E). These findings are consistent with the observed increase in the expression of G1/S transition genes (*CCND1*, *CCNE1*, *CCNE2*, *E2F2*, and *E2F3*) following the double overexpression of DPPA2/4 (Supplementary Materials Additional File S2: Figure S3F). Additionally, the analysis of apoptosis-related genes revealed a downregulation in the expression of pro-apoptotic genes (*BAX* and *BIM*), alongside an upregulation in the expression of the anti-apoptotic gene (*BCL-2*) in DPPA2/4^OE^ bEPSCs (Supplementary Materials Additional File S2: Figure S3G), providing additional evidence that DPPA2/4 potentially promote cell proliferation by regulating the G1/S transition. Therefore, our results showed that DPPA2/4 are a positive regulator of cell proliferation in bEPSCs.

Next, we investigated the potential of these cells to undergo in vitro differentiation. The ability of Control^OE^, DPPA2^OE^, DPPA4^OE^, and DPPA2/4^OE^ bEPSCs to form EBs was evaluated. As depicted in Supplementary Materials Additional File S2: Figure S4A, all the cells could form EBs. qRT-PCR analysis revealed significant differences in the mRNA expression levels of three germ layer marker genes (of the endoderm, mesoderm, and ectoderm) between the treated groups and the control group. Specifically, the mRNA levels of *GATA4* and *SOX17*, which are endoderm markers, and *GBX2* (a mesoderm marker) were decreased in DPPA2/4^OE^ EBs (Supplementary Materials Additional File S2: Figure S4B). Immunostaining using anti-AFP, anti-GFAP, and anti-SMA antibodies revealed differential expression of AFP, GFAP, and SMA proteins in the EBs of the different treatment groups (Supplementary Materials Additional File S2: Figure S4C). Hence, DPPA2/4^OE^ potentially delays early differentiation in bEPSCs. These results indicate that double overexpression of DPPA2/4 delays the differentiation of bEPSCs.

### 3.5. DPPA2/4 Activate the PI3K/AKT/GSK3β/β-Catenin Signaling Pathway to Affect the Pluripotency of bEPSCs

**Figure 6:**
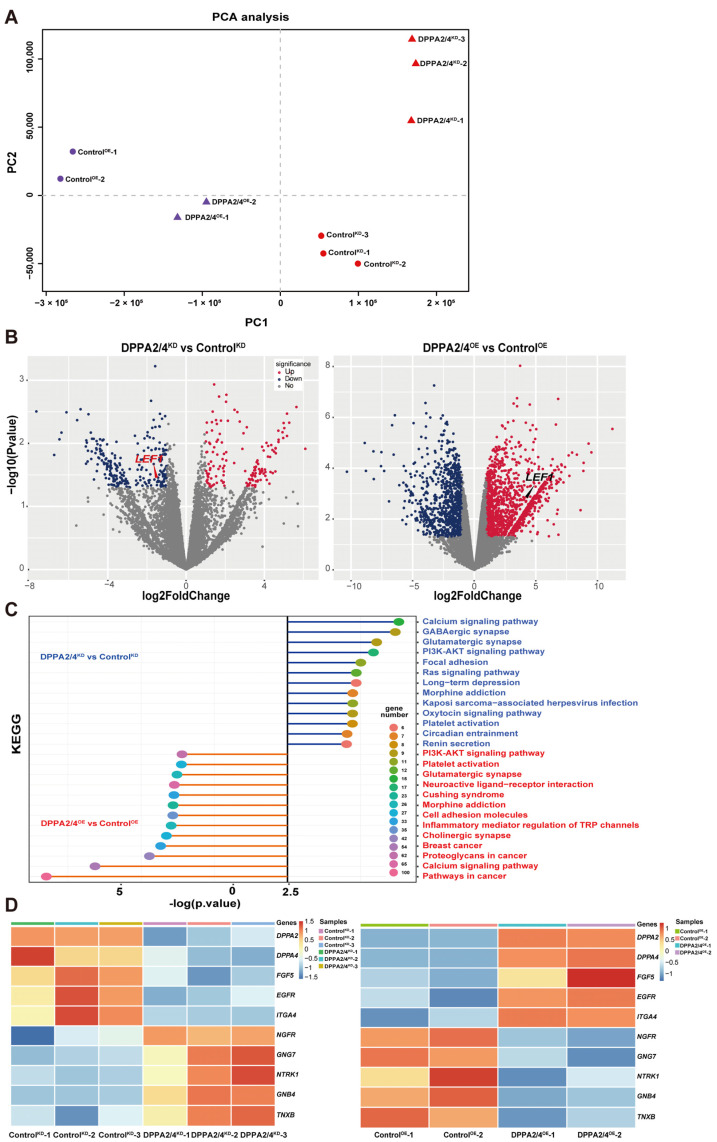
RNA-seq analysis revealed that DPPA2/4 affect the PI3K/AKT signaling pathway in bEPSCs. (**A**) PCA of DPPA2/4-knockdown and DPPA2/4-overexpressing RNA-seq datasets. (**B**) Volcano plot of the differentially expressed genes. (**C**) KEGG enrichment analysis of the differentially expressed genes. (**D**) Heatmap of PI3K/AKT pathway-related genes in Control^KD^, DPPA2/4^KD^, Control^OE^, and DPPA2/4^OE^ bEPSCs.

We next investigated the molecular mechanisms involved in the regulatory network of DPPA2/4 in bEPSCs. RNA-seq was applied to analyze the gene expression profiles of bEPSCs with DPPA2/4 double overexpression or knockdown. Principal component analysis of gene expression indicated distinct transcriptomic features in both the DPPA2/4 double overexpression and knockdown groups and the control group (Figure 6A). Notably, a total of 2238 genes and 400 genes exhibited differential expression following DPPA2/4 double overexpression and knockdown, respectively (Figure 6B). KEGG enrichment analysis of the DEGs associated with DPPA2/4 double overexpression or knockdown revealed that the PI3K/AKT signaling pathway was significantly enriched in both sets of DEGs (Figure 6C), and heatmap analysis revealed substantial differences in the expression levels of PI3K/AKT pathway genes between DPPA2/4-overexpressing and DPPA2/4-knockdown bEPSCs (Figure 6D), which suggested that DPPA2/4 may regulate the pluripotency and proliferation of bEPSCs by activating the PI3K/AKT pathway.

**Figure 7:**
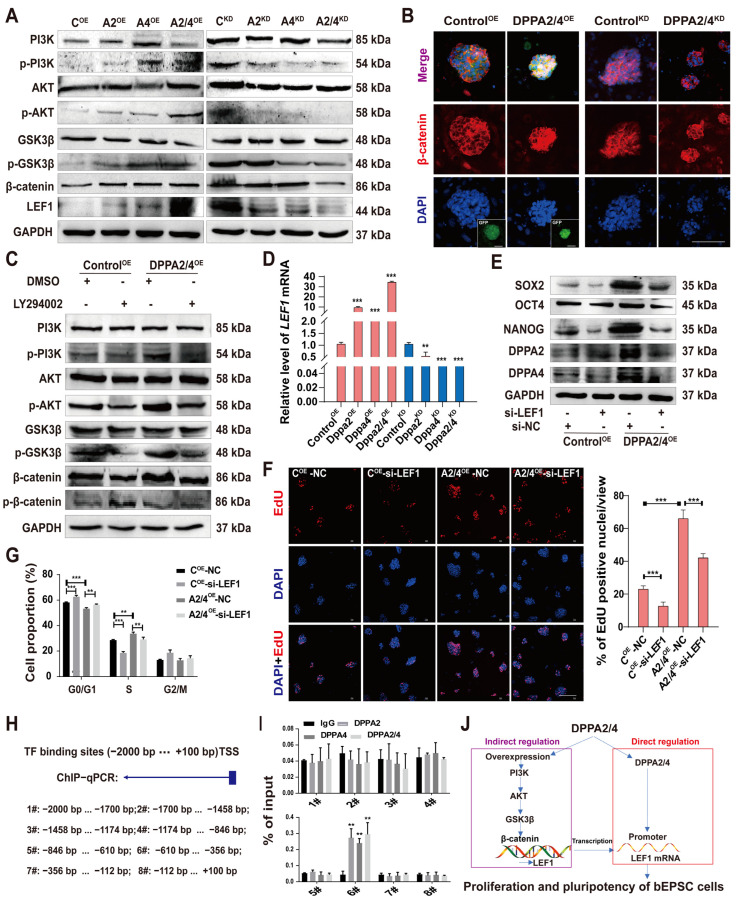
Induction of LEF1 by DPPA2/4 activates the PI3K/AKT/GSK3β/β-catenin pathway and maintains cell proliferation and pluripotency. (**A**) Western blotting analysis of p-PI3K, PI3K, p-AKT, AKT, p-GSK3β, GSK3β, β-catenin, and LEF1 protein levels in DPPA2/4 knockdown and overexpression bEPSCs. (**B**) β-catenin localization was detected by immunofluorescence staining with an anti-β-catenin antibody in Control^OE^, DPPA2/4^OE^, Control^KD^, and DPPA2/4^KD^ bEPSCs. Scale bars, 50 µm. (**C**) Western blotting analysis of p-PI3K, PI3K, p-AKT, AKT, p-GSK3β, GSK3β, p-β-catenin, and β-catenin protein expression in bEPSCs following treatment with the PI3K inhibitor LY294002. (**D**) qRT-PCR analysis of *LEF1* expression in different cell lines. (**E**) Western blotting analysis of OCT4, SOX2, NANOG, DPPA2, and DPPA4 protein expression in different treatment groups of bEPSCs. (**F**) Immunofluorescence images were obtained by the EdU incorporation (red, Alexa Fluor 555) assay. Scale bars, 20 µm. (**G**) Cell cycle analysis of LEF1-knockdown bEPSCs. The percentages of cells in different phases are indicated. (**H**) Prediction results of the binding of DPPA2/4 to the site upstream of the TSS of LEF1. (**I**) ChIP–qPCR was used to detect *DPPA2/4* binding to the *LEF1* promoter region in bEPSCs. The data are presented as the means ± SDs; n = 3 independent experiments ** *p* < 0.01; *** *p* < 0.001.). (**J**) The hypothesized model for the signaling pathways involved in DPPA2/4-induced bEPSC proliferation and pluripotency.

The PI3K/AKT signaling pathway plays an important role in regulating the survival and self-renewal of pluripotent stem cells and can jointly promote the proliferation and maintenance of the pluripotent state of ESCs via Wnt/β-catenin signaling [30,31]. Therefore, we performed Western blotting to measure the phosphorylation levels of related proteins. The results showed a decrease in the phosphorylation of PI3K, AKT, and GSK3β in DPPA2/4^KD^ bEPSCs (Figure 7A). The results of immunofluorescence staining further demonstrated that double overexpression of DPPA2/4 increased the accumulation of nuclear β-catenin (Figure 7B), suggesting that DPPA2/4 activate the PI3K/AKT/GSK3β/β-catenin pathway to regulate the proliferation and pluripotency of bEPSCs.

We then administered a PI3K inhibitor to Control^OE^ and DPPA2/4^OE^ bEPSCs and assessed the expression of proteins such as p-PI3K, PI3K, p-AKT, AKT, p-GSK3β, GSK3β, p-β-catenin, and β-catenin (Figure 7C). The results showed that the PI3K inhibitor treatment significantly decreased the levels of p-PI3K, p-AKT, p-GSK3β, and β-catenin. Moreover, the increases in p-PI3K, p-AKT, p-GSK3β, and β-catenin expression induced by DPPA2/4 double overexpression were reversed by treatment with the PI3K inhibitor LY294002. Our findings indicate that DPPA2/4 modulate the pluripotency and proliferation of bEPSCs by engaging the PI3K/AKT/GSK3β/β-catenin pathway.

### 3.6. DPPA2/4 Exert Their Effects through LEF1 via the PI3K/AKT/GSK3β/β-Catenin Pathway and through Direct Binding to the LEF1 Promoter

LEF1 is an important Wnt signaling effector that responds to Wnt signaling through stable binding with β-catenin to the promoters of Oct4 and Nanog, the expression of which are regulated to maintain the undifferentiated state of ESCs [32,33,34]. Our RNA-seq data revealed that LEF1 was among the 291 overlapping DEGs found in analysis of DPPA2/4-overexpressing and DPPA2/4-knockdown bEPSCs (Figure 6C). LEF1 was upregulated in DPPA2/4-overexpressing bEPSCs and downregulated in DPPA2/4-knockdown bEPSCs. qRT-PCR and Western blotting confirmed these results (Figure 7A,D).

Next, we investigated whether DPPA2/4 promote bEPSC proliferation and pluripotency by upregulating LEF1 expression. We used a siRNA to downregulate LEF1 in DPPA2/4-overexpressing bEPSCs (Supplementary Materials Additional File S2: Figure S5A,B). The results showed that LEF1 knockdown hindered the ability of DPPA2/4 to induce the expression of OCT4, SOX2, and NANOG (Figure 7E). Interestingly, the decrease in LEF1 expression did not affect the expression of DPPA2/4 in bEPSCs (Figure 7E). These results suggest that the stimulatory effect of DPPA2/4 on pluripotency and proliferation specification is, at least partially, a result of the upregulation of LEF1.

Moreover, to determine whether LEF1 activity was required for the proliferation of DPPA2/4^OE^ cells, siRNA-LEF1 was transfected into DPPA2/4^OE^ cells. EdU and cell cycle experiments showed that LEF1 knockdown inhibited the DPPA2/4 double overexpression-mediated promotion of bEPSC proliferation (Figure 7F,G, Supplementary Materials Additional File S2: Figure S5C). In addition, double overexpression of DPPA2/4 increased the expression of cell cycle markers and apoptosis-related genes, which was reversed by LEF1 knockdown (Supplementary Materials Additional File S2: Figure S5D,E).

DPPA2/4 function as DNA-binding proteins; they can directly associate with the promoter region of a gene, consequently controlling its transcription [35]. Therefore, we investigated whether DPPA2/4 bind to the *LEF1* promoter region and directly regulate its transcription. We designed primers upstream and downstream of the starting site of the *LEF1* gene (−2000 to +100). ChIP-PCR was performed on bEPSCs (Figure 7H). The results showed that both DPPA2 and DPPA4 were recruited to the region of the *LEF1* promoter (#6) (Figure 7I), which suggested that DPPA2/4 directly regulate LEF1 expression by binding to the promoter region of *LEF1* in EPSCs.

## 4. Discussion

DPPA2 and DPPA4 (DPPA2/4) are nuclear proteins capable of heterodimerization, and they have been used as markers of pluripotency in mouse and human cells [8,9]. DPPA2/4 serve as transcriptional regulators in both pluripotent and cancer cells. Many studies have utilized these genes as markers of successful reprogramming due to their highly selective expression pattern in pluripotent stem cells [36,37]. DPPA2/4 also serve as a trigger of signaling pathways to promote zygote genome activation by binding to CG-rich regions [35]. Several studies indicate that the single or double knockout of Dppa2/4 has limited impacts on mESCs, and most Dppa2/4 double knockout mice can survive the initial stage of embryo development. Significant defects become apparent in the later stage of development, which result in the death of numerous knockout pups due to lung and skeletal abnormalities [11,14]. Additionally, they play significant roles in modulating the transitions to and from pluripotency [14,15,28,38,39]. However, the precise role of DPPA2/4 in the reprogramming of bovine fibroblasts has not been determined. In this study, to enhance our understanding of the molecular mechanism of EPSCs, we conducted an analysis of chromatin properties (ATAC-seq) and gene expression (RNA-seq) in EPSCs and fetal fibroblasts (FFs) from public databases and identified the EPSC-associated differentially expressed genes *DPPA2* and *DPPA4*. The regulatory effects of these two proteins were subsequently investigated through a series of experiments, including overexpression, knockout, knockdown, and rescue experiments, which were coupled with transcriptional analyses during the establishment and maintenance of bovine EPSC pluripotency. We showed that DPPA2/4 play an integral role in controlling the transition to pluripotency. Furthermore, DPPA2/4 can promote the pluripotency and proliferation of bEPSCs by regulating the G1/S phase transition during the process of pluripotency maintenance. It has been discovered that in DPPA2 knockout embryonic stem cells (ESCs), the expression of pluripotency-related genes can still be sustained, while their proliferative capacity is reduced [40,41]. Here, depletion of DPPA2/4 accelerated the early differentiation of bEPSCs, potentially as a consequence of the absence of DPPA2/4 during the pluripotency stage. Our study deepens the understanding of the role of DPPA2/4 in PSCs.

The RNA-seq data revealed enrichment of differentially expressed genes in the PI3K/AKT pathway when DPPA2/4 were double knocked down or overexpressed in bEPSCs. Our findings suggest that DPPA2/4 primarily regulate the proliferation and pluripotency of bEPSCs through modulation of the PI3K/AKT signaling pathway. The significance of PI3K/AKT signaling in the proliferation and maintenance of ESC self-renewal has been widely acknowledged [42,43,44]. Previous studies have reported that specific activation of the PI3K/AKT pathway is necessary for inducing cyclin D1 and facilitating the transition from the G1 to S phase in ESCs [43,45]. It has also been documented that PI3K activation upregulates NANOG, which is both mediated by and contributes to the maintenance of ESCs, while inhibiting the PI3K/AKT pathway leads to the loss of pluripotency marker expression [46,47,48]. In light of these data and based on the results of our study, we conclude that DPPA2/4 modulate the transcription of genes in the PI3K/AKT pathway and enhances the phosphorylation of multiple proteins, such as p-PI3K, p-AKT, and p-GSK3β; moreover, DPPA2/4 can increase the nuclear accumulation of β-catenin. These results suggest that DPPA2/4 promoted bEPSC pluripotency and proliferation via activation of the PI3K/AKT/GSK3β/β-catenin pathway.

LEF1, a transcription factor belonging to the TCF/LEF family, is characterized by the presence of an HMG domain. Its regulatory role in the maintenance of undifferentiated ESCs has been established through its regulation of OCT4 and NANOG [34]. Moreover, studies on human colon cancer cells have shown that MYC activates the expression of LEF1, thus promoting cell proliferation [49], and that LEF1 also plays a pivotal role in regulating lineage differentiation in pluripotent stem cells [32,33]. According to our RNA-seq analysis, after DPPA2/4 were double knocked down, we observed a significant decrease in LEF1 expression, whereas its expression significantly increased upon DPPA2/4 double overexpression. The activation of GSK3β is known to be necessary for β-catenin accumulation [50]. After GSK3β is phosphorylated at Ser9, it is inactivated by p-AKT [51,52]. Our study revealed that DPPA2/4 decreased GSK3β activity by phosphorylation, and the accumulation of nuclear β-catenin further increased. Subsequent experiments illustrated that DPPA2/4 increase LEF1 expression via the PI3K/AKT/GSK3β/β-catenin pathway, in turn influencing the pluripotency and proliferation of EPSCs. Intriguingly, we also discovered that DPPA2/4 directly bind to the promoter region of *LEF1*, thereby promoting its expression (Figure 7J).

The findings from human and mouse ESCs in addition the results of our study support the idea that the processes involved in the maintenance of self-renewal, pluripotency, and cell cycle machinery are intricately interconnected and work together to maintain PSC identity. Collectively, our findings highlight previously unrecognized aspects of DPPA2/4 and provide new mechanistic insights into their role in sustaining the undifferentiated state of bEPSCs. Understanding how DPPA2/4 function will be crucial for future basic research and for the safe utilization of bEPSCs.

There is a limitation in this article, as the bEPSCs^C^ and bEPSCs^A2/4^ obtained through reprogramming show that the endogenous OSKM remains highly expressed after activation, while the exogenous OSKM gradually becomes silenced. However, it is undeniable that in the bEPSCs^C^ and bEPSCs^A2/4^, some exogenous pluripotency genes have not been completely silenced. The residual expression of exogenous genes may be due to the strong CAG promoter.

## 5. Conclusions

In conclusion, our study highlights the role of DPPA2/4 in the reprogramming of bEPSCs and in maintenance of pluripotency of bEPSCs. DPPA2/4 promoted the proliferation capability and pluripotency of bEPSCs through activation of the PI3K/AKT/GSK3β/β-catenin signaling pathway and subsequent activation of the LEF1 transcription factor. These findings shed light on the involvement of DPPA2/4 in the establishment and maintenance of EPSCs in bovines, laying the foundation for developing a comprehensive theoretical framework for bovine embryonic stem cells.

## Data Availability

The datasets used and analyzed during the current study are available from the corresponding author upon reasonable request.

## Supplementary Materials

The following supporting information can be downloaded at https://www.mdpi.com/article/10.3390/cells13050382/s1, Additional File S1, Cell-specific genes in EPSCs and FFs; Additional File S2, Figure S1. Detection of the expression of endogenous and exogenous transcription factors after reprogramming. Figure S2: DPPA2/4 knockdown affects the pluripotency and early differentiation of bEPSCs; Figure S3: Overexpression of DPPA2 and DPPA4 increases the pluripotency of bEPSCs and promotes their proliferation; Figure S4: DPPA2/4 overexpression delayed bEPSC differentiation; Figure S5: DPPA2/4 promote bEPSC proliferation and pluripotency by upregulating LEF1 expression; Table S1: The detailed information for each dataset; Table S2: The sgRNA sequences targeting DPPA2 and DPPA4; Table S3: The primers used for the DPPA2/4 CDS amplification; Table S4: The siRNA sequences for DPPA2, DPPA4, and LEF1; Table S5: The primers used in the qRT-PCR analyses; Table S6: The primers used in the ChIP-qPCR analyses.

## Abbreviations

DPPA2/4	Developmental pluripotency-associated 2/4
LEF1	Lymphoid enhancer-binding factor 1
bEPSCs	Bovine expanded potential pluripotent stem cells
KSR	Knockout serum replacement
LIF	Leukemia inhibitory factor
MEF	Mouse embryonic fibroblasts
CDK	Cyclin-dependent kinases
GO	Gene Ontology
KEGG	Kyoto Encyclopedia of Genes and Genomes
qRT-PCR	Quantitative real-time polymerase chain reaction
ChIP	Chromatin immunoprecipitation
CDS	Coding sequences
TSS	Transcription start site
