# Supplementary material for: DPPA2/4 Promote the Pluripotency and Proliferation of Bovine Extended Pluripotent Stem Cells by Upregulating the PI3K/AKT/GSK3β/β-Catenin Signaling Pathway
Source: Cells. 2024 Feb 23;13(5):382. doi: 10.3390/cells13050382 (PMC10930381; doi:10.3390/cells13050382)
Supplement: Supplementary file 1 [file cells-13-00382-s001.zip › Additional file S2.pdf]

DPPA2/4 promote the pluripotency and proliferation of bovine extended pluripotent stem cells by upregulating the PI3K/AKT/GSK3β/β-catenin signaling pathway

Shu Fang[1], Jing Wang[1], Guangbo Liu[1], Burong Qu[1], Jian Chunyu[1], Wenqiang Xu[1], Jinzhu Xiang[1]*, Xueling Li[1]*

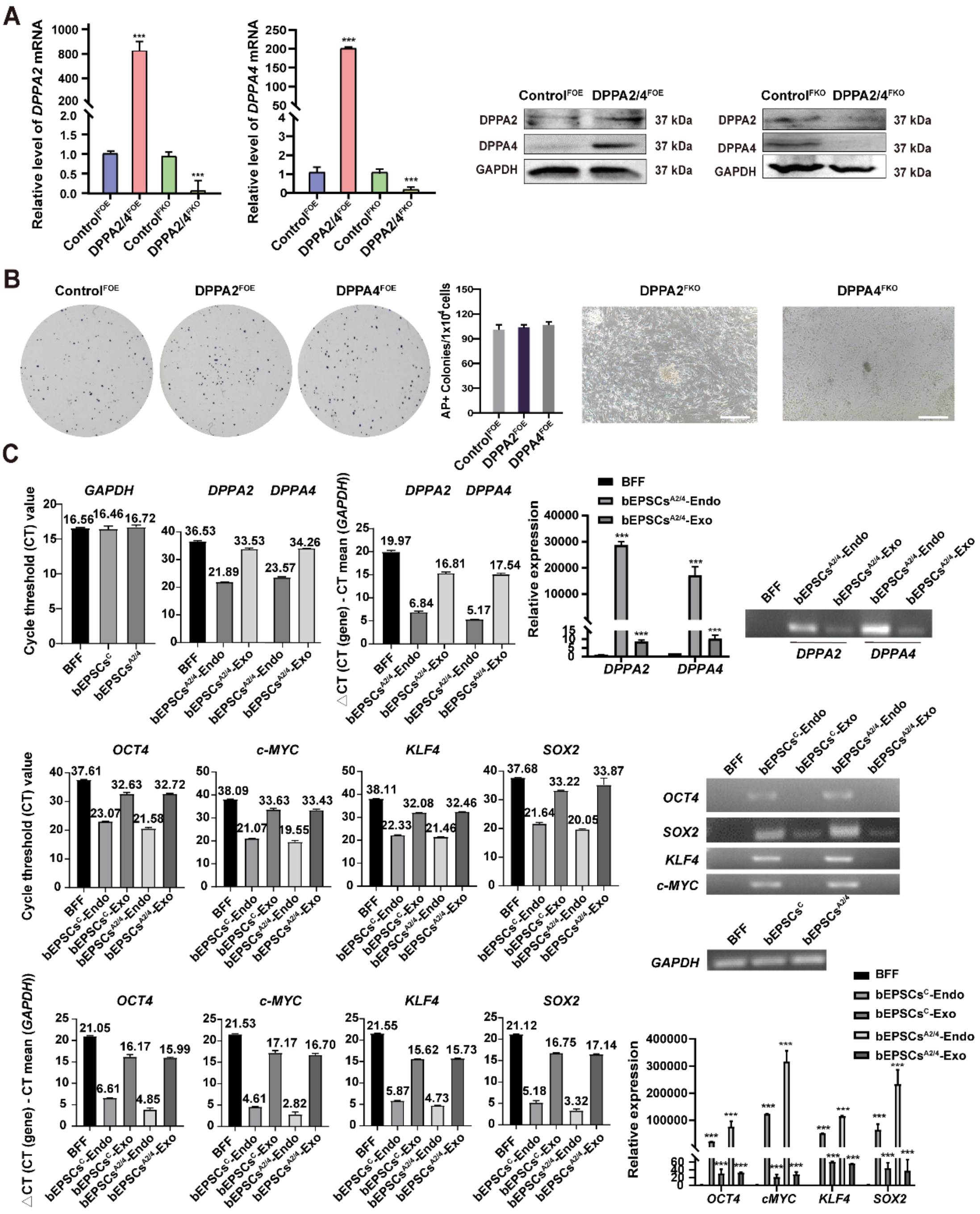

Figure S1. Detection of the expression of endogenous and exogenous transcription

factors after reprogramming. **(A)** qRT–PCR and Western blotting analysis of the expression of DPPA2 and DPPA4 in the control, DPPA2/4^{OE}, and DPPA2/4^{KO} bESCs. **(B)** AP staining of reprogrammed BFFs from the control, DPPA2 or DPPA4-single overexpressing, and DPPA2 or DPPA4-single knockout groups. **(C)** qRT–PCR analysis of endogenous and exogenous genes (*OCT4, SOX2.c-MYC*, *KLF4, DPPA2* and *DPPA4*) (left), Agarose nucleic acid electrophoresis picture of endogenous and exogenous genes (*OCT4, SOX2.c-MYC*, *KLF4, DPPA2* and *DPPA4*) (right).

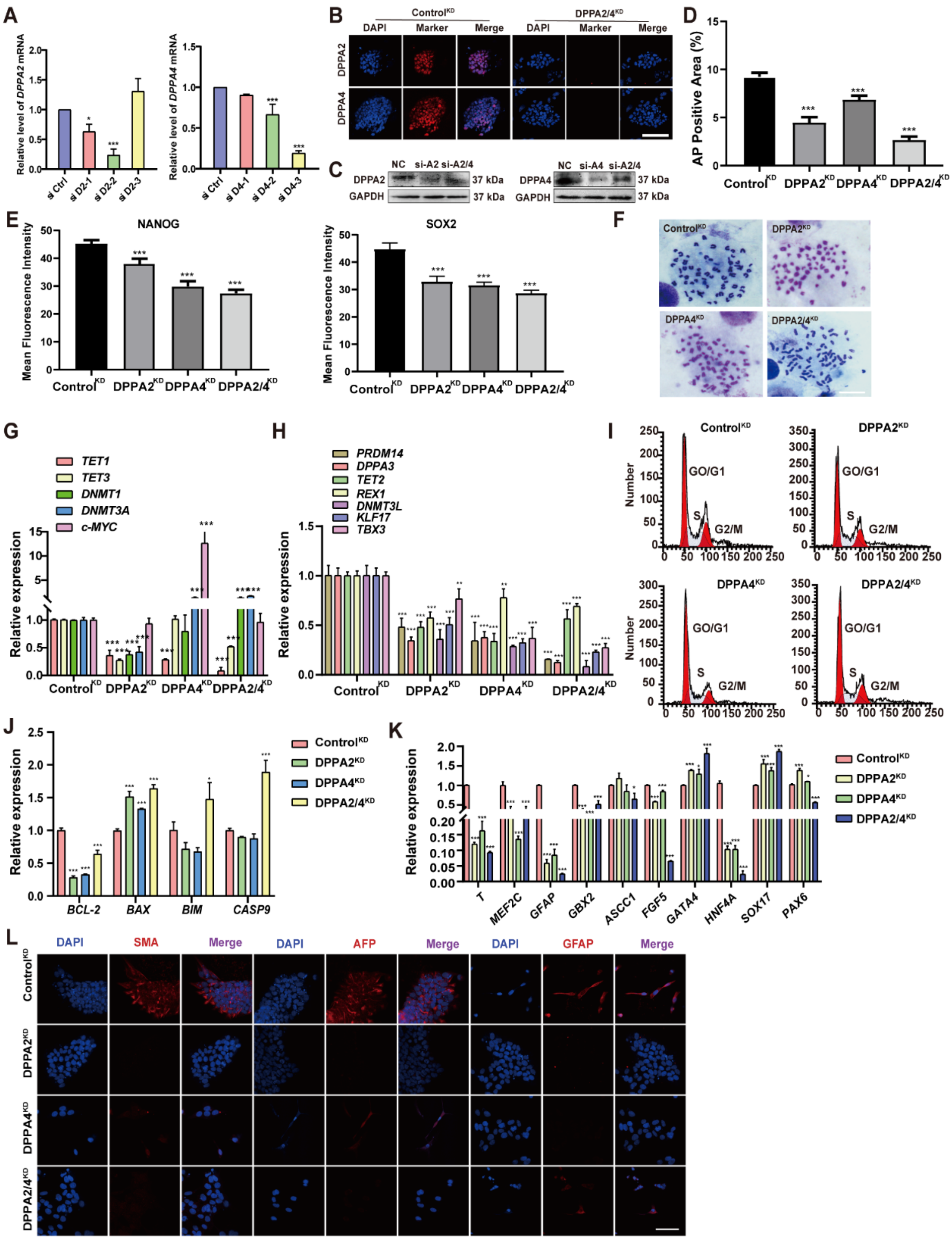

Figure S2. DPPA2/4 knockdown affects the pluripotency and early differentiation of bEPSCs. (A) qRT–PCR analysis of *DPPA2* and *DPPA4*. **(B)** Immunostaining of DPPA2 and DPPA4 in Control^{KD} and DPPA2/4^{KD} bEPSCs. Scale bar, 50 μm. **(C)** Western blotting analysis of DPPA2 (left) and DPPA4 (right) in Control^{KD}, DPPA2^{KD}, DPPA4^{KD}, and DPPA2/4^{KD} bEPSCs. **(D)** AP positive area (%) in Control^{KD}, DPPA2^{KD}, DPPA4^{KD}, and DPPA2/4^{KD} bEPSCs. **(E)** Mean fluorescence intensity of NANOG and SOX2 in Control^{KD}, DPPA2^{KD}, DPPA4^{KD}, and DPPA2/4^{KD} bEPSCs. **(F)** Karyotype analysis of Control^{KD}, DPPA2^{KD}, DPPA4^{KD}, and DPPA2/4^{KD} bEPSCs. Scale bar, 20 μm. **(G)** qRT–PCR analysis of the primed marker genes. **(H)** qRT–PCR analysis of the naïve marker genes. **(I)** Cell cycle analysis of Control^{KD}, DPPA2^{KD}, DPPA4^{KD}, and DPPA2/4^{KD} bEPSCs by flow cytometry. The percentages of cells in different phases are indicated. The representative histograms presented here show the distribution of cells in sequential phases (G0/G1; S; and G2/M) of the cell cycle. **(G)** qRT–PCR analysis of apoptosis-related genes. **(K)** qRT–PCR analysis of EBs at day 20. **(L)** Immunofluorescence staining for AFP, GFAP, and SMA. Scale bars, 50 μm. The data represent the means ± SDs; n = 3 independent experiments (* $p < 0.05$; ** $p < 0.01$; *** $p < 0.001$).

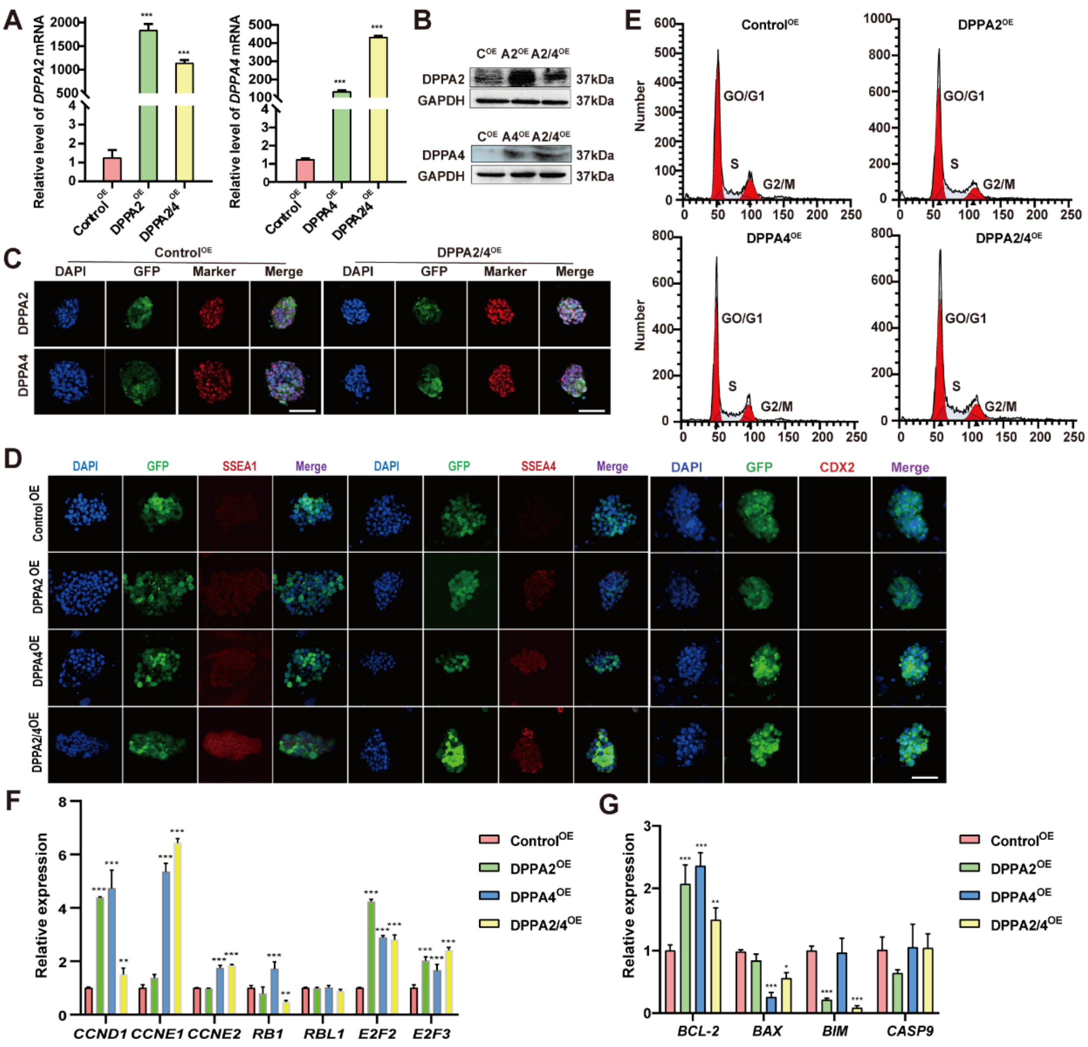

Figure S3. Overexpression of DPPA2 and DPPA4 increases the pluripotency of bEPSCs and promotes their proliferation. (A) qRT–PCR analysis of *DPPA2* and *DPPA4*. **(B)** Western blotting analysis of DPPA2 and DPPA4 in Control^{OE}, DPPA2^{OE}, DPPA4^{OE}, and DPPA2/4^{OE} bEPSCs. **(C)** Immunostaining of DPPA2 and DPPA4 in Control^{OE} and DPPA2/4^{OE} bEPSCs. Scale bar, 50 μm. **(D)** Immunofluorescence staining for SSEA1, SSEA4, and CDX2. Scale bars, 50 μm. **(E)** Cell cycle analysis of Control^{OE}, DPPA2^{OE}, DPPA4^{OE}, and DPPA2/4^{OE} bEPSCs. **(F)** qRT–PCR analysis of cell cycle regulatory genes. **(G)** qRT–PCR analysis of apoptosis-related genes. The data are presented as the means ± SDs; n = 3 independent experiments (* $p < 0.05$; ** $p < 0.01$; *** $p < 0.001$).

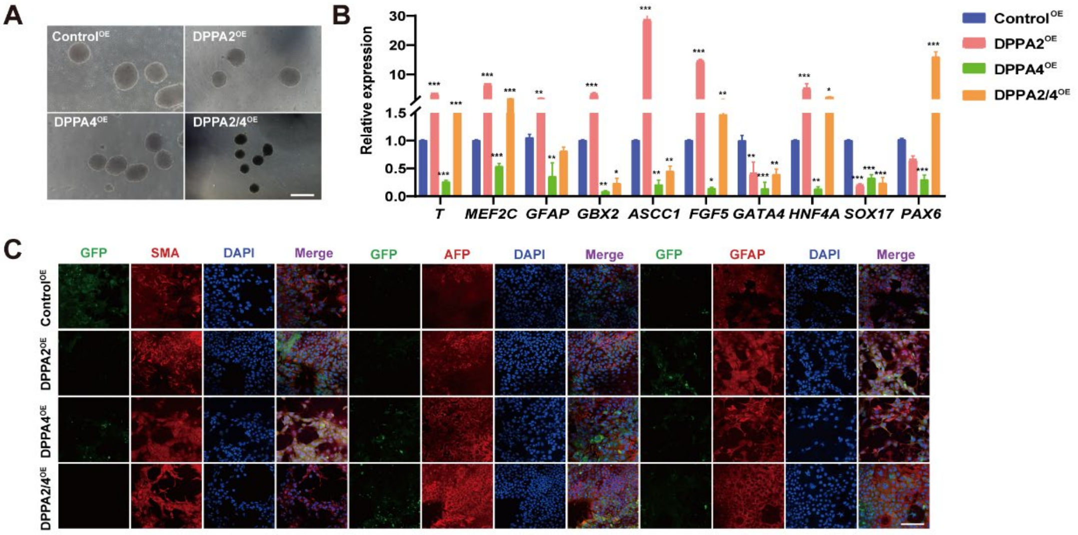

Figure S4. DPPA2/4 overexpression delayed bEPSC differentiation. (A) EB morphology of bEPSCs after DPPA2 and/or DPPA4 overexpression. Scale bars, 100 µm. (B) qRT–PCR analysis of EBs. (C) Immunofluorescence staining for AFP, GFAP, and SMA (n = 3). Scale bars, 100 µm. The data are presented as the means ± SDs; n = 3 independent experiments (* $p < 0.05$; ** $p < 0.01$; *** $p < 0.001$).

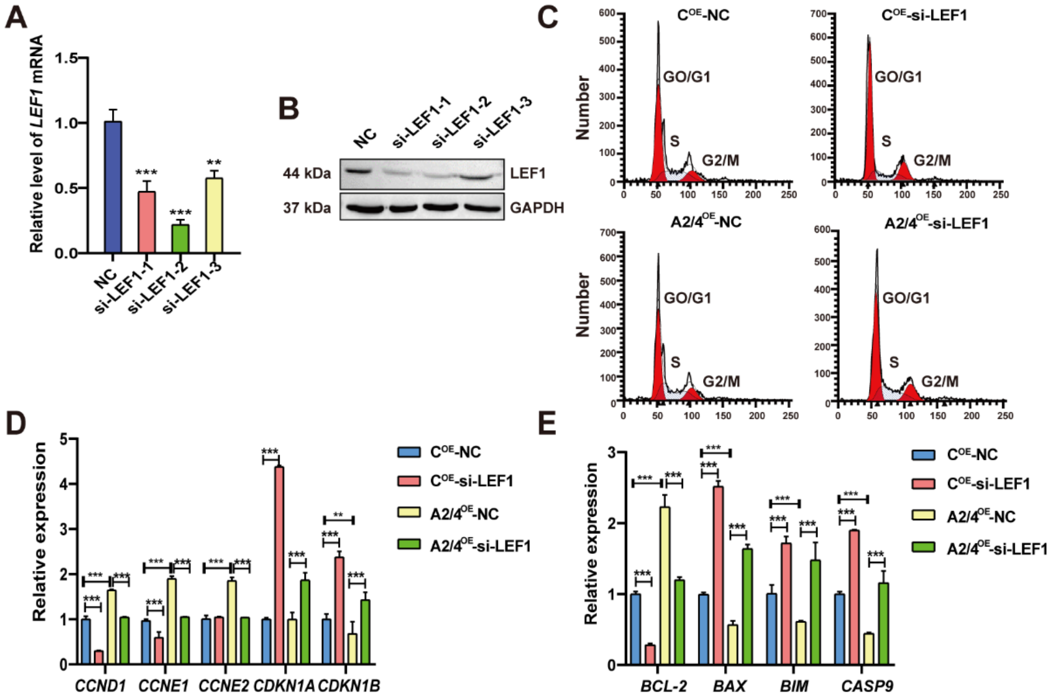

Figure S5. DPPA2/4 promote bEPSC proliferation and pluripotency by upregulating LEF1 expression. (A) qRT–PCR analysis of *LEF1* gene expression. **(B)** Protein levels of LEF1 in bEPSCs. **(C)** Cell cycle analysis of C^{OE}-NC, C^{OE}-si-LEF1, A2/4^{OE}-NC, and A2/4^{OE}-si-LEF1

bEPSCs. The percentages of cells in different phases are indicated. The representative histograms presented here show the distribution of cells in sequential phases (G0/G1; S; and G2/M) of the cell cycle. **(D)** qRT–PCR analysis of cell cycle regulatory genes. **(E)** qRT–PCR analysis of apoptosis-related genes. The data are presented as the means ± SDs; n = 3 independent experiments (* $p < 0.05$; ** $p < 0.01$; *** $p < 0.001$).

Table S1. The detailed information for each dataset.

GEO accession	Samples	Type
PRJNA693452	3 bovine EPSCs[1]	RNA-seq
GSE147839	2 human EPSCs	RNA-seq
GSE80732	2 mouse EPSCs	RNA-seq
GSE110036	2 bovine FFs[2]	RNA-seq
GSE176381	1 human FFs	RNA-seq
GSE127927	2 mouse FFs	RNA-seq
GSE157237	2 human EPSCs	ATAC-seq
GSE178323	2 human FFs	ATAC-seq

[1] EPSCs: expanded potential stem cells. [2] FFs: fetal fibroblasts.

Table S2. The sgRNA sequences targeting DPPA2 and DPPA4

Gene name	Primer sequence
sgDPPA2-1#	5′- ATGTGAGTCGGGATACTTTG-3′
sgDPPA2-2#	5′- AAATGGCATACTCAAATTAC-3′
sgDPPA2-3#	5′- TCTGTAGTGGGTATGTGCGT-3′
sgDPPA4-1#	5′- GGGATCTGCGTCGGTGTCTT-3′
sgDPPA4-2#	5′- GCGATCGCAAGAGACTTGTG-3′
sgDPPA4-3#	5′- GGGCTGCTCCTCCTGCACGG-3′

Table S3. The primers used for the DPPA2/4 CDS amplification

Gene name	Forward primer	Reverse primer
DPPA2	TGGCTCTGCCTTTCTC	ACGCACATACCCACTAC
DPPA4	TATCAGCCTTGACCTTT	TGCCTATAAGACCCATT

Table S4. The siRNA sequences for DPPA2, DPPA4, and LEF1

Gene name	Primer sequence

siDPPA2-1#	5'- CUCAACUUCACAAGUCAAATT-3'
siDPPA2-2#	5'- CCAAGUUCUAGCUUUAGAATT-3'
siDPPA2-3#	5'- CCUCCGAUUAACGAUGUGATT-3'
siDPPA4-1#	5'- GUUGAAAUUACAGGGAGAATT-3'
siDPPA4-2#	5'-CCAAAUGUGAUAGCAUCAATT-3'
siDPPA4-3#	5'- GUGAAACGAUCCUUCUGAATT-3'
siLEF1-1#	5'- GCCGACAUCAAGUCUUCCUTT-3'
siLEF1-1#	5'- GGACCCUCUUACUCGAGUUTT-3'
siLEF1-3#	5'- GCUACAUAUGCAGCUCUAUTT-3'

Table S5. The primers used in qRT-PCR analyses.

Gene name	Forward primer	Reverse primer
b-DPPA2	GTCGGGATACTTTGCGGAAC	CCATGGGATCACAGAGTCGT
b-DPPA4	TGAAACCTCCGAAGACACCG	CAGCCCCTTCAGAAGACGTT
b-OCT4	GGTTCTCTTTGGAAAGGTGTT	ACACTCGGACCACGTCTTTC
b-SOX2	CATCCACAGCAAATGACAGC	TTTCTGCAAAGCTCCTACCG
b-NANOG	TTCCCTCCTCCATGGATCTG	ATTTGCTGGAGACTGAGGTA
b-CDX2	CTCCTGGACAAGGACGTGAG	ACATGGTATCCGCCGTAGTC
b-DNMT3L	ATGAGCAACTGGGTCTGCTT	GGGCTCTCTCTTCCACACAG
b-DNMT3A	CTGGTGCTGAAGGACTTGGGC	CAGAAGAAGGGGCGGTCATC
b-DNMT1	AGTGGGGGACTGTGTTTCTG	TGTACGAGAGCTGCATGTCC
b-KLF4	TCCCACCGCTCCATTAC	ATGAGAACTCTTCGTGTAGG
b-KLF17	CCCTTCCCAGATGACTTTCA	GTGGGACATCATTGGGATTC
b-STELLA	TGCAAGTTGCCACTCAACTC	TTCCTTTGGCATAGCGAAGT
b-TET1	GAAACCGGAACCAATCTGTCC	GCTTCCTCTTTCTGAAAACCC
b-TET2	ATGCCAGTTTGCAAAATTCATTC	GGGCTTCCATTCTGGAGCTT
b-TET3	GGAAGCGGTGTGGTACTTGT	GCTGAGCTCTGAGCCTGTCT
b-T	TGCTGAAGGTGAACGTGTCT	CACGATGTGGATTCGAGGCT
b-MEF2C	CAGCACCAACAAGCTGTTCC	ACCAAACTGTTGTGGCTGGA
b-GFAP	CCTGCAGATCCGAGAAACCA	TCCACGGTCTTCACCACAAT
b-GBX2	GCGAGGTGCAGGTGAAAATC	CCCCTGTCTTGGAATTGGCA

b-BMP4	GGCTGGAAGAAAAACAGAGTCC	ATGGCACTACGGAATGGCTC
b-ASCC1	TTCTGGATCCTTGGCCTGTAT	CTCCACGAGGCCTTGGTAAT
b-FGF5	GTACGTGGCCCTGAACAAGA	GTGGGTAGAGACGTGCTGAG
b-GATA4	GAGATGCGCCCCATCAAAAC	TAAGGCCAGGCTGTTCCAAG
b-HNF4A	CGGAATCAACGGCGACATTC	AAGGCTGGGATGTACTTGGC
b-SOX17	CCCCTCGGGGACATGAAGAT	TCAGTGCCTTCCACGACTTG
b-PAX6	GAATTCTGCAGGTGTCCAACG	GTCTGATGGAGCCAGTCTCG
b-LEF1	TTCTAGGCAGAAGGTGGCAT	GCAGCTGTCATTCTTGGACC
b-MYC	CCCATCAGCACAATTACGCA	TGTCCGCCTCTTGTCATTCT
b-PRDM14	CGGAGACAATTCCCTGATGT	CACGGGAATGTCCAGAAACT
b-REX1	GGAAGAGGACCCACTCCTTC	ACTTGGCCTCCTAGTGCATC
b-DPPA3	GCAATGCAAGTTGCCACTCA	ATCCTGTACCTCCGCTCACT
b-KLF5	GCGGGTGCTGACCATGAG	GGTCTACGACTGAGGCACTG
b-TBX3	CTGCTACTGGGGAACAGTGG	GGAGTTCAGTATAGTAAATCCGTGC
b-BCL-2	ATGTGTGTGGAGAGCGTCAA	GGGCCATACAGCTCCACAAA
b-BAX	GCCCTTTTGCTTCAGGGTTT	GTCCAATGTCCAGCCCATGA
b-BIM	TACCAGATCCCCGCTCTTCA	CGTAGCTCTTGGGCAATCCA
b-CASP9	GATCAGGCCAGGCAGCTAAT	CGGCTTTGATGGGTCATCCT
b-CCND1	GCACTTCCTCTCCAAGATGC	GGTTGGAAATGAACTTCACG
b-CCNE1	GTCCCCTGACCATTGTGTCC	ACACCACTGATACCCTGAAACC
b-CCNE2	CGGGTCTGGCGAGGTTT	AGGCGGCCAACAATTCCTTA
b-E2F2	GGAGGACAAGGCCAACAAGA	TGCCTCTGGTTGTTGAGAGG
b-E2F3	CCTGGAGCAGTACCTGGTGA	CCGCTTTCTCCTAGCTCCAG
b-E2F4	GAAGACCTGCTCCAGAACCC	GCCTCCACTCACTGGAATCTC
b-E2F7	AAGTGCACGTCACGGAAGAT	GGAGTAGACTCCTGTGGAGC
b-CDKN1A	GAGACCGTGGTTGGGAGAC	AAAGTCGAAGTTCCACCGCT
b-CDKN1B	AGGACACGCATTTGGTCGAT	TTCTGAGGCCAGGCTTCTTG
b-RB1	CTGTGTGCACGCTTTCTGTC	GTCTCCTGAACAGCATGAGGAA
b-RBL1	CAAGAATGGGTCGGGCCTTA	AACTCTTAGAAGGGCTGCCG
b-GAPDH	GGGTCATCATCTCTGCACCT	GGTCATAAGTCCCTCCACGA

Table S6. The primers used in ChIP-qPCR analyses.

Gene name	Forward primer	Reverse primer
1# '-2000 to -1700	CAGTCACTAAGTCGTGT	GCAAATAGGTGAGAATG
2# '-1687 to -1458	GCTTTACTCTCTCTGGCTCC	TCAAAATATCGCTTATTGGT
3# '-1458 to -1174	GTGCTCGTTTAGAGCAT	TTTGTTCCTTGGCTCTC
4# '-1174 to -846	TTTCACAAAGCATCTCC	AGGTCGCTCGTGTATTTTCG
5#'-819 to -610	TGAGGATGTAATGAAAAAGA	GGAAGAAGATGAAAGAGAAG
6#'-576 to -367	GAGAGGCGGAAAAGAGGTGG	TAGGGGGAAGATTCAGGCAA
7#'-356 to -112	GCGCGTACACACACACA	GCTGAAAAGCTGCCCAC
8#'-103 to +98	GGCGGCGGGAGGAGGAGAGG	CCGCAGAGCGCACAGCCTGG
